# Advances in Analytical Methods for the Extraction and Quantification of Benzophenones in Breast Milk and Infant Formula: A Scoping Review and Bibliometric Analysis

**DOI:** 10.3390/foods15101693

**Published:** 2026-05-12

**Authors:** Marcella Vitoria Galindo, Danyelly Silva Amorim, Isabelly Silva Amorim, José Teixeira Filho, Wellington da Silva Oliveira, Helena Teixeira Godoy

**Affiliations:** 1 Department of Food Science and Nutrition, University of Campinas (UNICAMP), Campinas 13083-862, SP, Brazil; d265723@dac.unicamp.br (D.S.A.); i235277@dac.unicamp.br (I.S.A.); helenatg@unicamp.br (H.T.G.); 2 Faculty of Agricultural Engineering, University of Campinas (UNICAMP), Campinas 13083-875, SP, Brazil; joseteix@unicamp.br

**Keywords:** human milk, infant formula, benzophenones, analytical methods, mass spectrometry, bibliometric analysis

## Abstract

Benzophenones (BPs) and derivatives are endocrine-disrupting chemicals (EDCs) widely used in personal care products, food packaging, and flavoring ingredients. This systematic review and bibliometric analysis aimed to identify and summarize analytical methods used to determine BPs in human milk and infant formulas. Furthermore, the bibliometric evaluation explored publication trends by journal, citation count, and geographical distribution, providing insight into the global research landscape on this topic. The most employed sample preparation techniques included liquid–liquid extraction, solid-phase extraction, dispersive solid-phase extraction, low-temperature partitioning, QuEChERS, and dispersive liquid–liquid microextraction, frequently combined with enzymatic treatments with β-glucuronidase or arylsulfatase to improve recovery and sensitivity. Gas chromatography (GC) and liquid chromatography (LC) coupled with mass spectrometry (MS) were the predominant analytical platforms, with LC–MS being the most used for its ability to detect BPs without derivatization. Recent studies have shown a trend of replacing conventional organic solvents with greener, sustainable, and environmentally friendly approaches, such as miniaturized methods. This trend aligns with Green Analytical Chemistry principles and highlights the need for ongoing methodological and regulatory advancements to ensure food safety and protect public health.

## 1. Introduction

### 1.1. Benzophenones: Chemistry and Application

Benzophenones (BPs) are aromatic ketones composed of two aromatic rings linked by a carbonyl group, which may have different substituents that change their properties (Table 1). Derivatives of BPs are formed by inserting chromophore groups, thereby modifying their reactivity and functional properties. Benzophenone-1 (BP-1) to -12 (BP-12) are derivatives corresponding to 2-hydroxybenzophenone, in which hydroxy, methoxy, and methyl groups, among others, are added to the molecule (Table 1) [1]. BPs are solid at room temperature, soluble in organic solvents, and insoluble in water [2]. The log K*_ow_* of BPs ranges from 0.4 for BP-4 to 4.1 for BP-7. These compounds exhibit high boiling points (up to 500 °C), which results in low volatility. Furthermore, due to their lipophilic characteristic, BPs can persist for several thousand hours in the environment [3,4,5].

The widespread use of BPs is attributed to their broad-spectrum UV-absorbing capacity, consistent performance, and relatively low cost [7]. For UV filters, use is limited to a concentration of up to 6%. In the food packaging industry, BPs have been used as photo initiators for UV-cured printing inks and varnishes, and as additives. For personal care products, such as shampoos, skin lotions, makeup, and nail polishes, BPs might also be added to protect product formulation [8,9,10].

The scientific and regulatory communities have shown increasing interest in the environmental contamination caused by BPs. Water, soil, sediments, sludge and biota, food matrices, as well as in biological samples, including human milk (HM), have already been contaminated by BPs. Special attention has been given to BP-3, a high-production-volume chemical [7].

With regard to the HM, although products containing UV absorbers are formulated for topical application, researchers have noted that BP can be absorbed through the skin and metabolized, potentially leading to bioaccumulation in various parts of the body, including breast milk [11]. Consequently, there is increasing demand for the development of reliable and rapid analytical methods with sufficient sensitivity to determine BPs in diverse matrices at trace concentrations [12].

Several studies in the literature confirm the presence of BP and its derivatives in HM and infant formulas (IFs), with BP, BP-1, BP-2, BP-3, 4-OH-BP, BP-12, MBB, 4-PBZ, 4-MBP, DMPA, and HCPK being the most reported compounds [13,14,15].

### 1.2. Metabolism and Risks

The metabolism of xenobiotics, such as benzophenones, is mainly carried out in the liver by cytochrome P450 enzymes. Depending on the lipophilicity and solubility of the compound formed during metabolism, it may be excreted in urine or feces, or it may enter the intracellular space via diffusion, where it is transported by the blood to other organs of the body [16]. For BP, metabolism occurs in two phases to increase solubility and facilitate excretion. The first phase oxidizes the compound, alters its physicochemical properties, and provides active sites on the molecule that will be involved in phase II of the process. In phase II, conjugation (sulphation or glucuronidation) of the molecules occurs to promote the elimination via urine [8,16,17]. Some BPs, such as BP-1, BP-2, and 4-OH-BP, can be formed during the metabolism of BP-3. These compounds may be more harmful than BP-3 itself.

The absorption and metabolism of BPs may result in some adverse health effects for both mothers and infants, such as carcinogenic or endocrine effects [18]. BPs in the hydroxylated form, such as BP-3, BP-1, and BP-2, showed higher estrogenic activities in vitro and studies [19,20]. Furthermore, the presence of BP-1 in the urine of US and Chinese people is correlated with an increased risk of developing reproductive system diseases [21].

In humans, high levels of BP-3 exposure are associated with an unusual change in birth weight and gestational age in both girls and boys [22]. Schlumpf et al. [23] demonstrated that BP-3 exerts a uterotrophic effect in vivo and stimulates the proliferation of MCF-7 breast cancer cells. Studies carried out by Broniowska et al. [24] in rats suggest that BP-2 alters thyroid hormones.

The main route of baby exposure to BP-3 is through dermal contact, given that lotions, oils, and creams used on babies can contain up to 0.25% BP [25]. On the other hand, it is well known that HM may be a pathway for maternal excretion of environmental chemicals, including BP-1, BP-3, and 4-OH-BP. However, data on the incidence of BPs in breast milk are limited, even considering the potential short-, medium-, and long-term adverse effects of early exposure to these compounds on infant health, such as hormonal imbalance and obesity [26].

### 1.3. Legislation

The International Agency for Research on Cancer (IARC) classified BP as possibly carcinogenic to humans (Group 2B) [27]. Previous studies have found no scientific evidence to identify other BPs as carcinogenic to humans.

Regulations aimed at reducing exposure to BPs have been applied in several countries. In the United States, the Food and Drug Administration (FDA) has banned the use of BPs in printing inks and food packaging materials; however, their use in cosmetics is still permitted [28]. In Hawaii, the use of BP-3 in sunscreens has been prohibited due to its association with coral reef degradation and the negative impacts on marine vegetation [29].

In Canada, BP is not authorized for use in food, as listed on Health Canada’s List of Permitted Food Additives; however, its use in cosmetics is permitted. To characterize the risk of benzophenones associated with short-term dermal exposure, regulatory agencies in Canada set the No Observed Adverse Effect Level (NOAEL) at 20 mg/kg bw/day, based on a 28-day oral toxicity study. This value applies to children, teenagers, and adults [30].

In the European Union, BP is permitted as a flavoring substance for use in food, as specified in Regulation (EU) No. 872/2012 [31]. In addition, BP can be used as an additive or processing aid in food contact materials, with a maximum specific migration limit (SML) of 0.6 mg/kg [32].

In the latest scientific opinion published by the European Food Safety Authority (EFSA), BP was assessed as safe for use as a food additive at levels set by the Panel on Food Additives, Flavourings, Processing Aids, and Materials in Contact with Food. The reported Tolerable Daily Intake (TDI) for BP was set at 0.03 mg/kg body weight per day [33].

In Brazil, the Brazilian National Health Surveillance Agency (ANVISA) adopts the European Union’s specific migration limit for BP (0.6 mg/kg). However, for some derivatives, such as BP-1, BP-12, and 2-hydroxy-4-n-hexyloxybenzophenone, the SML was set at 6 mg/kg [9]. Overall, no limit has been set for all BP.

### 1.4. Infants’ Exposure to BPs Through Human Milk and Infant Formula

HM is considered the gold standard in nutrition because it provides all the nutrients, growth factors, and antibodies required for babies’ development after birth. Therefore, it is recommended that babies be exclusively breastfed for the first six months of life and breastfeeding should be carried out for two years or more [34]. According to the United Nations Children’s Fund (UNICEF) database, globally, 41% of children aged 0 to 5 months are exclusively breastfed.

The composition of HM varies due to the mother’s physiological factors and periods of lactation. Due to this, HM can be classified into three different stages: colostrum (three to five days after birth) has higher amounts of proteins, antibodies, and less fat; transition milk (up to 15 days after birth) is rich in fats and carbohydrates, with lower amounts of proteins and prebiotics; mature milk (after 15 days) has higher amounts of lipids, carbohydrates, and micronutrients important for the healthy development of infants. The composition of mature HM reported by several clinical studies is presented in Table 2 [35,36,37].

Due to the chemical composition of HM, lipophilic chemical compounds, such as BPs, tend to accumulate in both adipose tissue and milk, hindering the complete excretion of these compounds [38,39].

Although reported accumulation levels are generally below established limits, HM can result in continuous low-level exposure of infants to chemicals [40]. While BPs in HM do not automatically imply harm, their ubiquity raises concerns due to potential endocrine-disruption effects. BP-3 (oxybenzone) demonstrates estrogenic activity and has been associated with reduced birth weight in males and shorter gestation [41], though its reproductive toxicity remains inconclusive [42].

Because of this, HM has become a non-invasive biomonitoring matrix for monitoring chemical contaminants, as recommended by the World Health Organization (WHO). Furthermore, infants are more vulnerable to toxic and harmful compounds present in HM due to exclusive breastfeeding and their immature physiological systems that are not yet fully developed to efficiently metabolize toxic substances.

For children who do not receive breast milk or in cases where the mother does not produce enough milk to feed the baby fully, IF becomes an alternative to breastfeeding [34]. The IF has been classified as a formula for newborns, a transition formula (from six months), and a growing-up formula (from twelve months). Further, IF can be commercialized as liquid, powdered, and ready-to-drink products [43,44].

The development and improvement of IFs have expanded over the years to meet the physiological and nutritional needs of some babies, such as those with allergies or who are unable to breastfeed. Currently, there are IFs based on goat’s milk, rice, lactose-free, gluten-free, and partially or extensively hydrolyzed protein, which allow for a correct and healthy diet for babies with the most diverse nutritional demands.

The concentration of nutrients, including proteins, carbohydrates, lipids, vitamins, and minerals, varies according to the specific type of IF, such as anti-reflux formulations, hypoallergenic formulas, and those supplemented with prebiotics and probiotics. The incorporation of these nutrients is intended to support optimal growth and development of the baby [45].

IFs can also contain chemical substances, such as BPs, resulting from contamination of raw material and/or migration from paints or varnishes used in packaging. External factors and improper handling of the product also help in the migration process. The incidence of BPs in IFs has already been reported in the literature [46]. Moreover, the search for BPs in food has grown since the 1990s, in response to consumers’ increasing demand for healthy, safe foods.

Moreover, the development of new detection systems, as well as different sample preparation methods, allowed for more robust quantification of these compounds, in increasingly lower concentrations (µg./kg or ng/kg). Conversely, the use of large volumes of solvents and time-consuming sample preparation techniques were replaced by methods based on the principles of green chemistry.

The development and validation of analytical methods for monitoring BPs in foods, especially HM and IFs, have evolved over time to achieve more sensitive protocols to mitigate human exposure to these compounds. Several extraction and cleaning techniques have been used to determine benzophenones in HM and IFs. Miniaturized sample preparation methods have been standing out as a consequence of the reduction in solvent consumption, long sample preparation times, and good compatibility with different lipophilic matrices [47,48,49,50,51].

Given the widespread presence of BPs and their potential adverse effects on the health of lactating mothers and infants, this review aims to highlight advances and trends in analytical methods for the extraction and quantification of these compounds in HM and IFs. Although reviews on the incidence of benzophenones in breast milk and IFs are available in scientific literature, this is the first specific bibliometric study on the topic, elucidating the main authors, institutions, and countries that have contributed to the literature, as well as the most cited articles, prominent journals, and the most relevant themes in the field.

## 2. Materials and Methods

A scoping review is a valuable approach for synthesizing information from heterogeneous and emerging literature that lacks prior systematic mapping [52,53]. This study adheres to the Preferred Reporting Items for Systematic Reviews and Meta-Analyses Extension for Scoping Reviews (PRISMA-ScR) guidelines to provide a transparent and reproducible methodological structure [54]. The research design is based on the frameworks proposed by Silva et al. [55] and Amorim et al. [56], which combine the breadth of a scoping review with the quantitative depth of a bibliometric analysis, thereby enhancing the comprehensiveness and replicability of the findings. No protocol was registered for this study.

### 2.1. Search Strategy

The dataset was collected by searching the Web of Science and Scopus databases, which were selected for their relevance and comprehensive coverage of scientific literature on BPs. The search was conducted in August 2025 and included all publications up to that year. It was utilized combinations of keywords and Boolean operators (AND, OR), incorporating terms related to breast milk, infant formula, and benzophenones, such as: “HUMAN MILK” OR “BREAST MILK” OR “MATERNAL MILK” OR “INFANT FORMULA” AND “BENZOPHENONE” OR “BENZOPHENONES” OR “2,4-DIHYDROXYBENZOPHENONE” OR “2,2′,4,4′-TETRAHYDROXYBENZOPHENONE” OR “OXYBENZONE” OR “2,2-DIHYDROXY-4,4-DIMETHOXYBENZOPHENONE” OR “DIOXYBENZONE” OR “2-HYDROXY-4-(OCTYLOX)BENZOPHENONE” OR “4-HYDROXYBENZOPHENONE” OR “METHYL-2-(BENZOYL)BENZOATE” OR “PHENYL BENZOPHENONE” OR “4-METHYLBENZOPHENONE” OR “2,2-DIMETHOXY-2-PHENYLACETOPHENONE” OR “1-HYDROXYCYCLOHEXYL PHENYL KETONE”.

### 2.2. Eligibility Criteria and Study Selection

**Figure 1:**
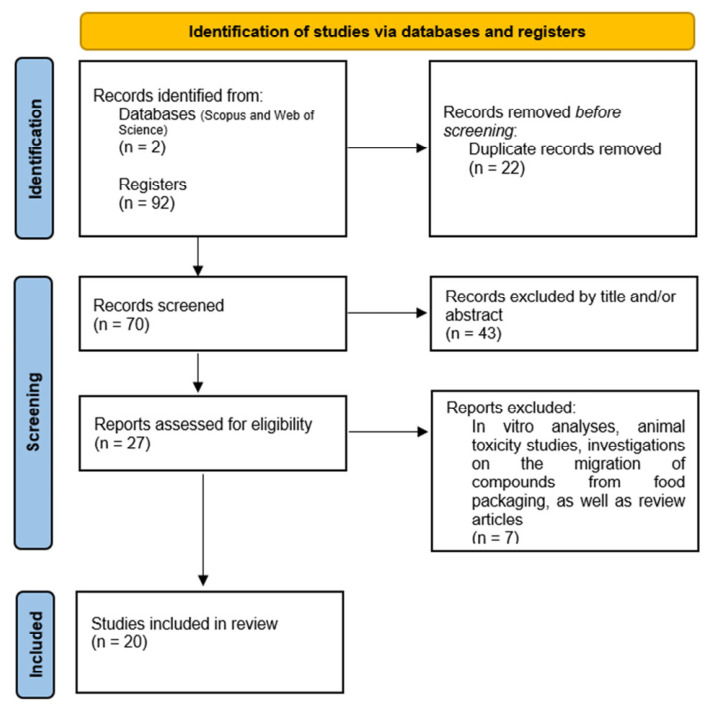
Prisma flow diagram.

According to the PRISMA-ScR reporting model, the study identification and selection process was conducted in sequential stages (Figure 1). Initially, 92 records were identified across the consulted databases (Scopus and Web of Science). After removing 22 duplicate articles, 70 unique records remained for the title and abstract screening phase. The initial screening of titles and abstracts was conducted independently by two reviewers, strictly following the pre-defined eligibility protocols. Any discrepancies between the evaluators were resolved through consensus during discussion meetings. When agreement was not reached, a third senior researcher was consulted to make the final decision.

During the eligibility stage, studies that did not meet the defined scope were excluded, including in vitro analyses, animal toxicity studies, investigations of compounds migration from food packaging, and review articles and book chapters. After full-text reading and detailed assessment, 20 documents were considered eligible and included in the qualitative synthesis. These 20 studies specifically applied analytical methods to detect BP in HM and IF, constituting the final set of methodological studies analyzed. Unlike the standard PRISMA guidelines for systematic reviews, PRISMA-ScR does not require a formal risk-of-bias or methodological quality assessment. In this scoping review, our primary objective was to map the technical landscape and advances in analytical methods used in the field, describing their technical approaches and applications. Thus, clinical outcomes were not synthesized, and considerations such as effect size estimation, reporting bias, certainty of evidence, and sensitivity analyses were not applicable.

### 2.3. Research Tools and Data Management

Following the selection process, full metadata were retrieved through targeted searches in the Scopus and Web of Science databases. The variables extracted included primary bibliographic identifiers (titles, publication years, and journals), authorship metadata (names, affiliations, and countries), content descriptors (author keywords and Keywords Plus), and citation counts and reference lists.

For bibliometric processing, data were exported as “.bib” file for use in Bibliometrix (version 4.5.0) and as “.txt” file for network modeling in VOSviewer (version 1.6.20). Analysis was performed using Biblioshiny, a web-based interface for the Bibliometrix R-package, which facilitated the import and visualization of bibliographic metrics [57]. To ensure data integrity, we assumed the accuracy of the records provided by the indexed databases.

Specific simplifications were applied to refine the dataset: (i) manual de-duplication and standardization of author names were performed to resolve phonetic or spelling variations, and (ii) a minimum occurrence threshold of five citations was set in VOSviewer to prioritize significant nodes in the bibliographic coupling networks. Subsequently, a theoretical synthesis of the primary research themes was conducted. Both platforms offer robust frameworks for comprehensive data interpretation [58].

## 3. Bibliometric Study Results

### 3.1. Characteristics of Scientific Publications

The systematic analysis resulted in 20 articles meeting the defined eligibility criteria, spanning 2006 to 2024 across 13 journals (Table 3). Additional details regarding the bibliometric results can be found in the Supplementary Material.

**Table 3:** Details of selected articles included in the review, showing the analytes evaluated, sample analytical methodology, instrument used, and the concentration detected in human milk or infant formula.

Compounds	Analytical Methodology	Instrumentation	Sample	LOQ/Recovery(%)/Calibration	Concentration	Reference
BP-3	Enzymatic treatment + LLE + on-line SPE	HPLC-MS/MS	Human milk (*n* = 4)	0.51 ng/mL/100/ Curve in water	<LOD—1.28 ng/mL	Ye, et al. [59]
BP-3	Enzymatic treatment + online SPE	HPLC–MS/MS	Human milk (*n* = 20)	0.4 ng/mL/100/ Curve in water	n.d—417 ng/g	Ye, et al. [60]
BP	LLE	HPLC-UV HPLC-TOF-MS	Infant formula (*n* = 16)	20 µg/kg/83/ Acetonitrile	n.d	Sanches-Silva, Pastorelli, Cruz, Simoneau, Castanheira and Paseiro-Losada [46]
BP-1, BP-2, BP-3, BP-6, BP-8, 4-OH-BP	LLE + protein denaturation	UHPLC-MS/MS	Human milk (*n* = 10)	0.9 ng/mL/100/ Matrix-matched	n.d—17.4 ng/mL	Rodríguez-Gómez, Jiménez-Díaz, Zafra-Gómez, Ballesteros and Navalón [14]
BP-1, BP-3, 4-OH-BP, BP-6, BP-8	protein denaturation + SBSE	GC–MS/MS UHPLC-MS/MS	Human milk (*n* = 10)	0.9 ng/mL/100/ Matrix-matched	(n.d—9.9 ng/mL) (n.d—10.2 ng/mL)	Rodríguez-Gómez, et al. [61]
BP-1, BP-3, 4-OH-BP, BP-6, BP-8	Solid–liquid extraction + UAE + dSPE	UHPLC–MS/MS	Human milk (*n* = 10)	0.9 ng/mL/100/ Matrix-matched	n.d—15.7 ng/mL	Rodríguez-Gómez, Zafra-Gómez, Dorival-García, Ballesteros and Navalón [50]
BP	QuEChERS + Captiva ND Lipids	GC-MS/MS	Human milk (*n* = 5)	0.4 μg/g/77 Matrix-matched	0.40—3.98 ng/g	Baduel, Mueller, Tsai and Gomez Ramos [47]
BP-3	Enzymatic treatment + on-line SPE	HPLC-MS/MS	Human milk (*n* = 9)	0.51 μg/L	<LOD—10.4 μg/L	Hines, Mendola, von Ehrenstein, Ye, Calafat and Fenton [49]
BP-1, BP-2, BP-3, BP-6, BP-8, 4-OH-BP	Enzymatic treatment + protein denaturation + DLLME	UHPLC–MS/MS	Human milk (*n* = 15)	0.5 ng/mL/100 Matrix-matched	n.d—1.5 ng/mL	Vela-Soria, Jiménez-Díaz, Díaz, Pérez, Iribarne-Durán, Serrano-López, Arrebola, Fernández and Olea [51]
BP-1, BP-3, BP-6, BP-8, 4-OH-BP	Enzymatic treatment + QuEChERS	UHPLC–MS/MS	Human milk (*n* = 15)	0.4 ng/mL/100 Matrix-matched	n.d—4.3 ng/mL	Vela-Soria, et al. [62]
BP-1, BP-3	Protein denaturation + on-line turbulent flow chromatography	HPLC-MS/MS	Human milk (*n* = 79)	0.1 ng/g/70 Matrix-matched	<LOD—799.9 ng/g	Molins-Delgado, et al. [63]
BP, 4-MBP, MBB, PBZ, DMPA, HCPK	LLE	UHPLC-MS/MS	Human milk (*n* = 60)	0.1 ng/mL/100 High ME	<MQL—1459 pg/mL	Liu and Mabury [13]
BP	LLE + LTP + GPC + SPE	GCxGC/TOF-MS	Human milk (*n* = 3)	-	n.d	Tran, et al. [64]
BP, BP-1, BP-3, BP-8, BP-12	LLE + LTP + dSPE	HPLC-DAD-FLD	Infant formula (*n* = 25)	0.1 mg/kg/100 Spiked matrix	n.d	Galindo, Oliveira and Godoy [48]
BP	Protein denaturation + SPE	UHPLC/MSMS	Human milk (*n* = 4)	1.41 ng/g/81 Matrix-matched	4 ng/g	Musatadi, et al. [65]
BP-1, BP-2 BP-3, BP-6, BP-8, 4-OH-BP	Enzymatic treatment + protein denaturation + DLLME + derivatization with BSTFA:ethyl acetate (60:40 *v*/*v*)	GC–MS/MS	Human milk (*n* = 83)	0.5 ng/mL/100 Matrix-matched	Percentile of 95% 0.19–2.21 ng/mL	Iribarne-Durán, et al. [66]
BP-1, BP-2, BP-3, BP-8, BP-12, 4-OH-BP	LLE + UAE + SPE	UHPLC/MSMS	Human milk (*n* = 65)	0.0027 ng/mL/100	19.5—3000 pg/mL	Sun et al. [15]
BP-3, BP-3-M2, BP-3-M4, BP-1, BP-3-M3, BP-4	Enzymatic treatment + UAE-DLLME	HPLC-DAD-FLD	Human milk (*n* = 3)	170 ng/mL/100/ MCR-ALS	n.d	Alcaraz, et al. [67]
BP	ASE + GPC+ SPE	GC-QTOF-MS	Human milk (*n* = 467)	External calibration	Mean range 483 ng/g lipid weight	Cheng, et al. [68]
BP-1, BP-3, 4-OHBP, BP-6, BP-8, BP	DLLME	GC–MS/MS	Human milk (*n* = 36)	0.3 ng/mL/100 Matrix-matched	Mean range 0.07—8.70 ng/mL	Castillero-Rosales, et al. [69]

Analytical methodologies—LLE: liquid–liquid extraction; SPE: solid-phase extraction; dSPE: dispersive solid-phase extraction; SBSE: stir bar sorptive extraction; UAE: ultrasound-assisted extraction; QuEChERS: quick, easy, cheap, effective, rugged and safe; DLLME: dispersive liquid–liquid microextraction; LTP: low temperature partition; GPC: gel permeation chromatography; ASE: accelerated solvent extraction; MCR-ALS: multivariate curve resolution-alternating least-squares. Instrumentation—HPLC-MS/MS: high-performance liquid chromatography tandem mass spectrometry; HPLC-UV: high-performance liquid chromatography with ultraviolet detector; HPLC-TOF-MS: high-performance liquid chromatography with time-of-flight mass spectrometry; UHPLC-MS/MS: ultra high-performance liquid chromatography.

**Figure 2:**
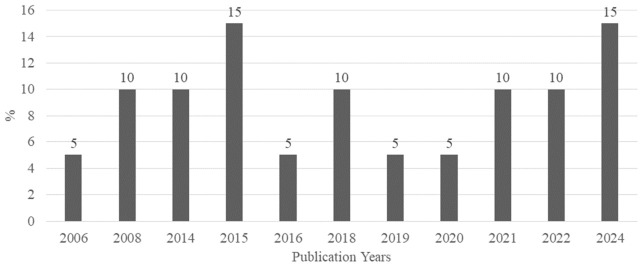
Evolution of publications on benzophenones in HM and IF by year.

The annual growth rate of publications was 6.29%, with an average document age of 7.8 years. The selected articles averaged 60 citations per document, totaling 762 references. Four publishers were identified, with Elsevier leading at 75%, followed by the American Chemical Society (15%), MDPI (5%), and Newlands Press Ltd. (5%). The 20 documents involved 110 authors, averaging 6.55 co-authors per article, with an international collaboration rate of 15%. Additionally, 74 distinct keywords were identified, reflecting the research’s thematic diversity.The number of annual publications is an indicator of the progress and interest in a given research topic [70]. Figure 2 presents the percentage distribution of publications over the years, with peaks in 2015 and 2024 (15%), lower reports in 2008, 2014, 2018, 2021, and 2022 (10% each), and minimal representation in the other years (5%).

**Figure 3:**
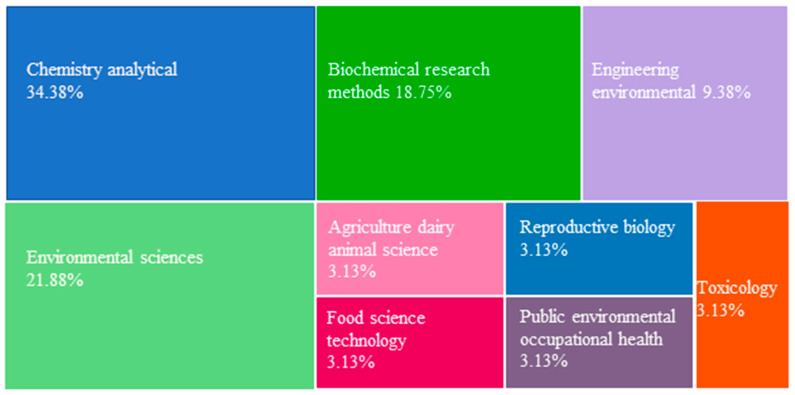
Research fields addressing the occurrence of benzophenones in human milk and infant formulas.

Regarding the areas, the Web of Science categories, the most representative fields were chemistry analytical (34.38%), environmental sciences (21.88%), and biochemical research methods (18.71%). Other areas include environmental engineering (9.38%) and fields such as toxicology, reproductive biology, and public health (3.13% each) (Figure 3), indicating a predominantly analytical and environmental focus on benzophenone detection and its impacts, alongside concerns about human health effects and maternal-infant exposure.

### 3.2. Journal Performance

Since 2006, research on benzophenones in HM and IF has been published in 13 distinct scientific journals. These sources are detailed in Table 4, categorized by publication volume and ranked according to Bradford’s Law of Scattering. This bibliometric principle is instrumental in identifying the “core” journals within a specific niche, based on the premise that a small group of high-productivity journals provides the most concentrated and relevant information for a given field [71].

Consequently, periodicals situated in Zone 1 are recognized as the primary outlets for the most significant contributions to this subject. Within this framework, the Journal of Chromatography A and Talanta emerged as the leading journals, publishing research on both fundamental and applied separation Science [47] and on advancements in pure and applied analytical chemistry [50].

### 3.3. Most Cited Articles

Citation analysis is an efficient technique for scientific mapping, grounded in the premise that citations represent intellectual links between publications. By quantifying citations volume, this method enables the identification of the most influential articles within a theme. Therefore, citation counts serve as a more objective and direct indicator of the impact and relevance of research within its field [70]. Table 5 presents the top 10 most-cited publications globally. A significant variation in the total number of citations (TC) was observed, ranging from 219 citations for the most influential article to 34 for the least cited.

The study published by Ye, Bishop, Needham and Calafat [60] presents significant findings regarding the development and validation of an on-line solid-phase extraction-high-performance liquid chromatography-tandem mass spectrometry (on-line SPE-HPLC-MS/MS) method with peak focusing for quantification of five parabens (methyl, ethyl, propyl, butyl, and benzyl parabens), triclosan, and six other environmental phenols, including bisphenol A (BPA) and BP-3, in milk. Thus, it provides valuable insights into potential human exposure to these compounds.

The validated method demonstrated good reproducibility (inter-day coefficients of variation ranging from 3.5% to 16.3%) and precision (recoveries ranging from 84% to 119% across four spiking levels). Methylparaben, propylparaben, triclosan, BPA, OPP, and BP-3 were detected in some of the tested samples. The free species of these compounds appear to be the most prevalent in milk.

A similar study, published by Ye, Kuklenyik, Needham and Calafat [59] was the second most-cited publication. In this work, the authors developed a highly sensitive method for analyzing environmental phenols and chlorinated organic chemicals in breast milk. BPA and BP-3 were detected in over 60% of the tested samples.

The third most-cited publication evaluated the concentrations of environmental phenols (bisphenol A (BPA), 2,4- and 2,5-dichlorophenol, benzophenone-3, triclosan) and parabens (methyl, ethyl, butyl, propyl) in the milk, urine, and serum of lactating women from North Carolina. The study reported that non-persistent chemicals were detected in the majority of urine samples (53–100%) and less frequently in milk or serum (ethyl, methyl, or propylparaben, benzophenone-3, and BPA were detected in breast milk) [49].

Overall, the studies reported relatively low concentrations of benzophenones in human milk, with maximum levels reaching 799.9 ng/g [63]. Moreover, most research has focused primarily on the development and validation of analytical methods, limiting the availability of data on infant exposure to these compounds through the consumption of HM or IF.

Most of the studies reported limits of quantification below 1 ng/mL. Moreover, calibration was typically performed using matrix-matched approaches to mitigate matrix effects. Reported recoveries were generally higher than 80%, reaching approximately 100% in more than 60% of the studies, highlighting significant methodological improvements in the field. In contrast, studies that did not achieve high recoveries were often focused on non-target analysis or on the simultaneous quantification of multiple contaminants, where overall method performance was prioritized over maximizing recovery for a specific class of compounds.

Although a gradual shift can be observed from method development toward exposure and risk assessment, there remains a significant lack of information regarding the toxicological implications and safety of chronic low-dose ingestion of benzophenones by infants. This knowledge gap hinders a robust evaluation of potential health risks and underscores the need for more integrative studies combining occurrence data with exposure assessment and toxicological endpoints.

### 3.4. Geographic Distribution, Institutions, Collaborative Networks, and Trends

**Figure 4:**
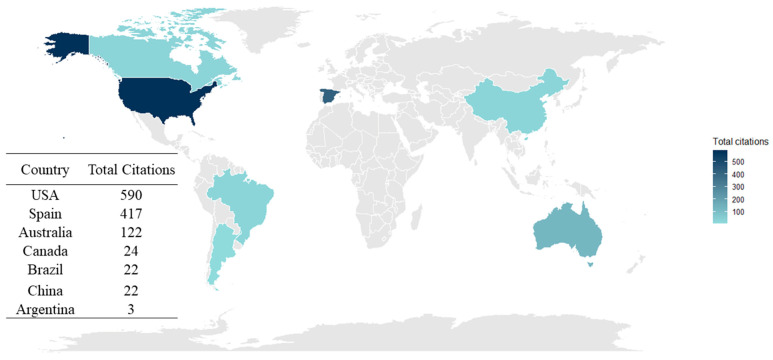
Most cited countries and average citations per article.

The global landscape of research on benzophenones in HM and IF is characterized by a strong concentration of scientific influence in a few key regions. The geographic distribution of citations identifies the United States as the primary leader with 590 citations, followed closely by Spain (417) and Australia (122) (Figure 4). While the U.S. maintains the highest absolute citation count, indicating a long-standing authority in the field, Spain emerges as a high-impact hub, particularly when cross-referenced with institutional data. Other nations, such as Canada, Brazil, and China, have contributed with a growing presence, though their total citation impact remains emerging compared to the established leaders.

**Figure 5:**
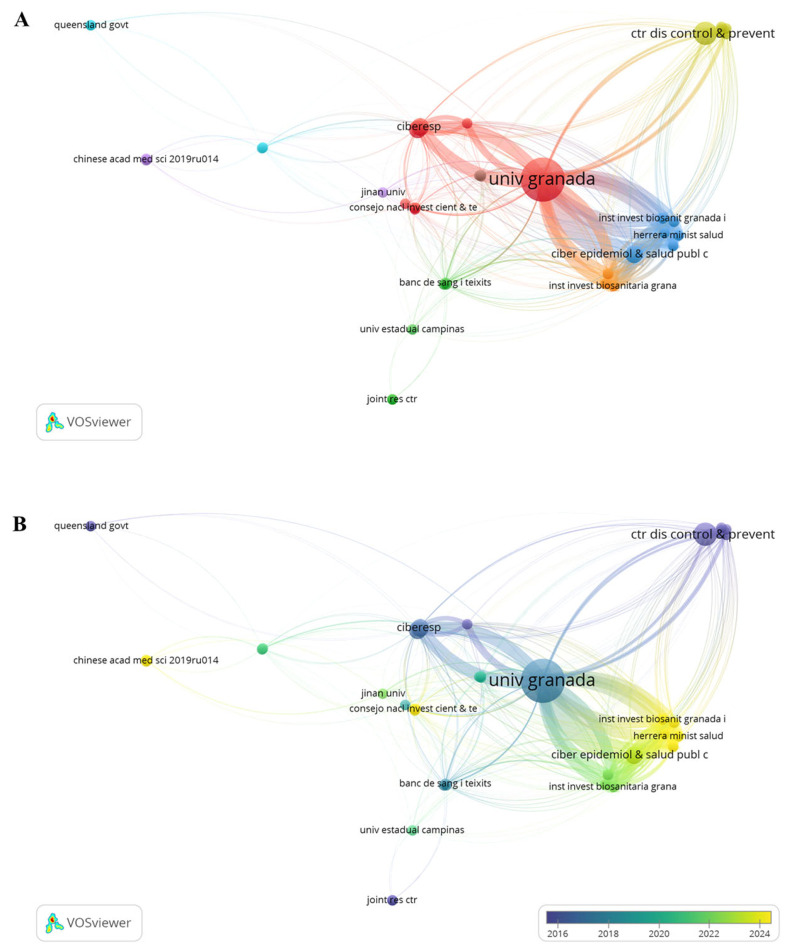
Bibliometric maps illustrating institutional collaboration. (**A**) Collaboration clusters are indicated by color, and publication volume is represented by node size. (**B**) Temporal trends are shown based on the average publication year.

The institutional collaboration network (Figure 5A) reveals a highly integrated core, where the University of Granada (Spain) [IA7.1] stands as a primary hub of the global network. The large node size for Granada reflects not only its high publication volume but also its strategic role in facilitating connections among different research clusters, such as those involving the Biomedical Research Networking Center for Epidemiology and Public Health (CIBERESP), which collaborates with the Department of Radiology and Physical Medicine. Furthermore, the Department of Analytical Chemistry at the same university has also conducted research on the development of analytical methods and on the monitoring of BP and other contaminants in HM. This Spanish cluster demonstrates robust internal synergy, likely driving the country’s high citation rates.

The temporal analysis (Figure 5B) provides a clear visualization of the field’s evolution from 2016 to 2025. Early research efforts (indicated by blue/purple nodes) were significantly anchored by institutions like the Centers for Disease Control and Prevention (CDC) in the U.S., focusing on foundational exposure assessments. Conversely, the more recent “yellow” frontier (2022–2025) shows an expansion towards Asian and Latin American institutions, such as the Chinese Academy of Medical Sciences, Jinan University, and the University of Campinas (Brazil). This shift suggests that while the methodological foundations were laid by North American and Spanish groups, the current research front is diversifying geographically, focusing on regional exposure profiles and emerging analytical challenges.

### 3.5. Keyword Analysis, Thematic Foci, and Future Trends

In bibliometric research, keyword co-occurrence analysis facilitates the identification of thematic clusters; when integrated with temporal analysis, it further elucidates emerging research trends and the evolution of prevalent topics over time [55].

**Figure 6:**
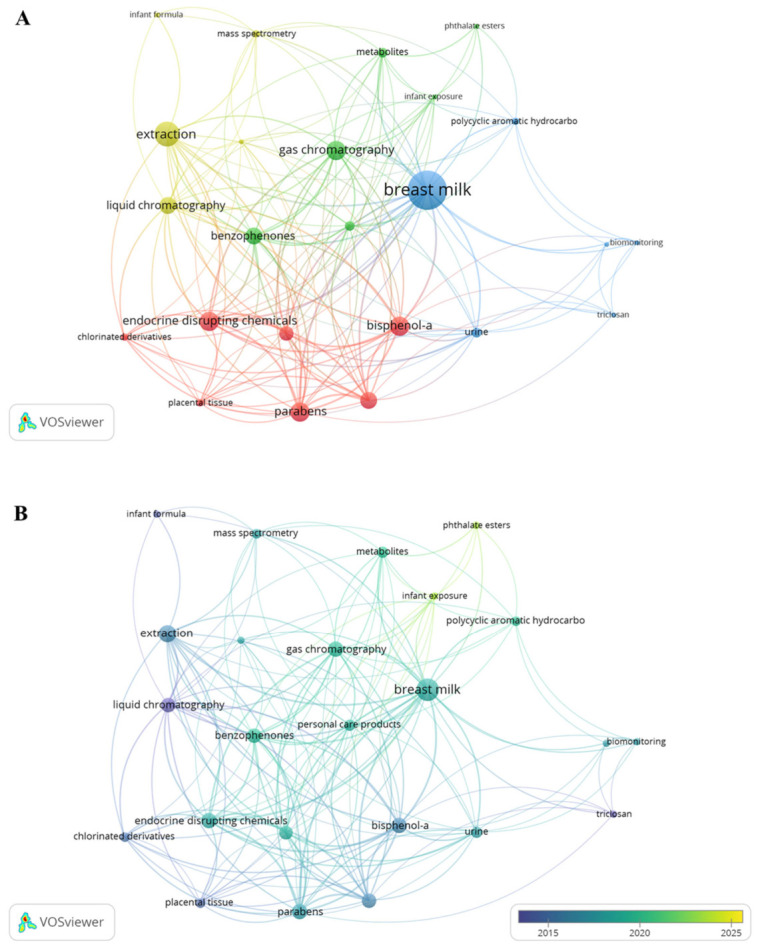
Bibliometric keyword maps. (**A**) Thematic clustering (colors) and frequency (node size). (**B**) Temporal trends based on the average publication year (2014–2025).

The analysis of thematic clustering and frequency (Figure 6A) demonstrates that research on benzophenones is well established in integrating analytical method development and contaminant monitoring. The term ‘breast milk’, identified as the primary central node of the network, connects directly to terms such as ‘extraction’ and ‘gas chromatography’, demonstrating a trend toward overcoming the complexity of this biological matrix through advanced analytical methodologies. Furthermore, the apparent proximity of benzophenones to other endocrine disruptors, such as bisphenol-A and parabens, indicates that these compounds are essential targets in neonatal biomonitoring studies.

The temporal evolution of the data between 2014 and 2025 (Figure 6B) indicates a clear maturation in the focus of publications. While the initial years of the analyzed period focused strictly on the technical validation of extractions and chromatographic methods, the most recent trend (2018–2020) points to a growing interest in ‘metabolites’ and ‘infant exposure’. This shift suggests that the field is transitioning from simple chemical detection toward the assessment of toxicokinetics and infant health risks, placing benzophenones at the center of contemporary discussions on chemical safety.

**Figure 7:**
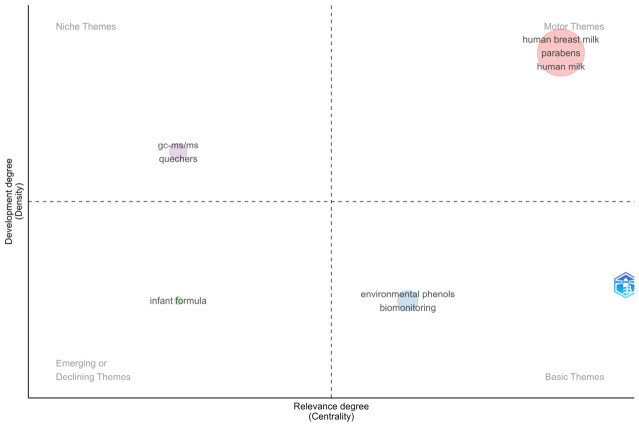
Conceptual mapping of the field: partitioning keywords into four sectors based on their integration and prominence.

The analysis of thematic maturity and relevance was structured using a strategic diagram, which categorizes topics into four quadrants based on their centrality and density (Figure 7). Centrality (*x*-axis) indicates the degree of interaction between a theme and the rest of the network, while density (*y*-axis) reflects its internal cohesion and stage of development. In this model, the upper-right quadrant houses motor themes, which are fundamental and well-consolidated in the field. Highly specific or specialized topics, known as niche themes, are located in the upper-left quadrant. Meanwhile, the lower-left quadrant identifies emerging or declining areas, whereas the lower-right quadrant groups basic themes, which possess high transversal relevance but still exhibit low theoretical density [56].

The strategic mapping highlights significant gaps, particularly regarding the diversification of study matrices. While breast milk represents a consolidated motor theme, ‘infant formula’ is classified as an emerging or underexplored theme, which justifies the need for this scoping review to fill this gap in the literature. Additionally, the positioning of methods such as QuEChERS and GC-MS/MS as niche themes indicates that, despite their robustness, these methodological advancements have not yet been fully integrated into routine monitoring, offering an opportunity to standardize analytical protocols for benzophenones.

**Figure 8:**
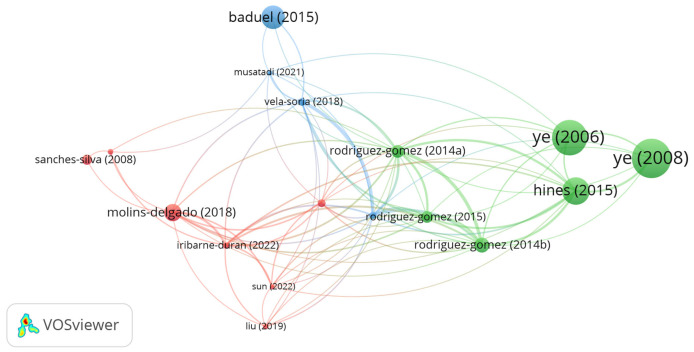
Cluster formation based on bibliographic coupling.

The interactions among different research groups were evaluated using co-authorship analysis. Figure 8 illustrates the bibliographic coupling network of articles with at least five citations. The nodes in the network represent articles, and the links between them represent bibliographic coupling.

Three distinct clusters were identified (Figure 8), each addressing specific subareas of the topic. Cluster 1 (red) focuses on the occurrence of organic contaminants in breast milk, such as bisphenol A and benzophenones, and on the development of highly sensitive analytical methodologies for their detection. Cluster 2 (green) investigates the presence of phenols and parabens in breast milk, emphasizing their potential endocrine-disrupting effects and employing advanced extraction and detection techniques, including ultrasound-assisted extraction and liquid chromatography. Cluster 3 (blue) focused on the development of strategies for the extraction and purification of contaminants in biological matrices utilizing dispersive liquid–liquid microextraction coupled with high-resolution mass spectrometry (LC-HRMS) or tandem mass spectrometry (LC-MS/MS). Overall, the cluster analysis highlights growing concerns regarding contamination in breast milk and reinforces the importance of robust analytical methodologies to monitor human exposure to these compounds.

The bibliometric analysis revealed the leading institutions and authors who have contributed most significantly to advancements in contaminant research, particularly in benzophenone determination. Understanding these key research themes helps focus this review on critical areas, including the chemistry of benzophenones, primary contamination pathways, and potential human health impacts, with particular attention to breast milk and infant formulas as sources of exposure.

## 4. Analytical Strategies Used for the Detection and Quantification of Benzophenones in Human Milk and Infant Formula

The determination of multiple residues in HM and IF presents an analytical challenge due to their complex composition, characterized by high lipid content, along with substantial amounts of carbohydrates, proteins, vitamins, minerals, and other minor constituents.

In general, extraction, cleaning, and/or concentration steps are required before instrumental analysis. The main objective of these steps is to obtain an extract with minimal matrix co-extractives, which might compromise the chromatographic process and, consequently, interfere with the detection of target compounds. Accordingly, the analyst must obtain prior information about the sample under investigation before selecting the extraction method and the analytical techniques for detecting the target compounds [47,72].

One of the primary challenges in extracting BPs from biological matrices is their frequent occurrence as conjugates with glucuronic acid and sulfate esters, which hinders the detection and quantification of their free forms. Several studies have reported the use of enzymatic treatments to address this issue and enhance the detection of BPs. In relation to the sample preparation methods, liquid–liquid extraction (LLE) and solid-phase extraction (SPE) were the most common analytical approaches used (Table 3).

Alternatively, techniques such as QuEChERS (Quick, Easy, Cheap, Effective, Rugged, Safe) [62], dispersive liquid–liquid microextraction (DLLME) [51], stir bar sorptive extraction (SBSE) [61], solid–liquid extraction (SLE) [50], low-temperature partitioning (LTP) [48,64], ultrasound-assissted extraction (UAE) [15,67], accelerated solvent extraction (ASE) [68], and dispersive solid-phase extraction (dSPE) have also been reported [48], both with or without combination with enzymatic processing.

All HM samples and IFs evaluated in this period required a solvent extraction step to isolate BPs from the matrices. Acetonitrile, acetone, hexane, and propanol were the most used solvents for extraction and clean-up [13,46,49,63]. Water was often added to dissolve the extract, which was further purified in an additional solvent-water partitioning step. Both LC and GC have been used to determine BPs in HM and IF. However, some BPs, such as 4-OH-BP, BP-1, BP-3, BP-8, BP-2, and BP-12, required a derivatization step with BSTFA solution prior to GC-MS analysis [66]. The addition of anhydrous salts (MgSO_4_ or NaCl) has been reported to separate the organic and aqueous phases, increasing the enrichment factor of BPs. Moreover, before the GC-MS analysis, MgSO_4_ was added to the final extract to remove residual water, thereby avoiding equipment-related issues.

This section provides a comprehensive overview of the main sample preparation methods and analytical instruments used to determine BPs in HM and IF, highlighting both their strengths and limitations. In addition, it will explore pre-extraction processes, such as enzymatic treatments and protein precipitation, emphasizing their critical role in enhancing analytical accuracy and reliability. Discussion on methodological standardization was not undertaken, as no official method is currently available for the determination of these compounds in HM or IF.

### 4.1. Pre-Extraction Processes

#### 4.1.1. Enzymatic Treatment

The use of enzymes such as β-glucuronidase and arylsulfatase has become increasingly promising for the sample preparation of biological fluids. The purpose of using the enzymatic solution containing β-glucuronidase and arylsulfatase prior to the chromatographic analysis is to cleave glucuronyl groups and sulfate esters, enabling the detection of the target molecules in both free and bound forms [59,62]. In the absence of reference standards for the conjugated metabolites, this enzymatic deconjugation step is critical to achieve adequate sensitivity and reliable quantification of the compounds.

The commercial enzymes reported were derived from *Helix pomatia*, with activities up to 463,000 U/g solid and were used at concentrations ranging from 60 to 460 U/mL. The enzymatic process involved mixing an aliquot of HM (100 µL–1 mL) with the enzyme solution (5–50 µL), followed by incubation at 37 °C for up to 24 h [49,59,60,62,66,67]. Native or isotope-labeled substrates, such as ^13^C4-4-methylumbelliferone, 4-methylumbelliferyl sulfate, and 4-methylumbelliferyl glucuronide, have been used to quantify the extent of the deconjugation reaction [60]. The use of enzymes was reported in 35% of the articles published in the last 24 years.

#### 4.1.2. Protein Denaturation

Protein denaturation has been widely used as a sample preparation technique for food, environmental, and biological samples, prior to chromatographic analysis. Protein removal has been achieved by adding acidic or basic solutions, organic solvents such as methanol, acetone, or acetonitrile, and inorganic salts. The addition of the precipitant acts directly on the protein surface, displacing the hydrophobic region, decreasing the protein–protein interaction, and decreasing solubility, leading to precipitation. Subsequently, the precipitated material can be removed by centrifugation or filtration [73,74]. In HM and IF, this technique was applied in 30% of the studies. Vela-Soria et al. [51] used acetone to precipitate proteins from milk samples. Rodríguez-Gómez et al. [14] also performed the HM protein and lipid extraction procedure using a solution containing zinc and tungsten salts. Both authors state that this procedure was excellent for cleaning and obtaining clear samples for subsequent steps. The simplicity and cost-effectiveness of using this technique before analyzing milk and other foodstuffs have been highlighted. However, protein precipitation does not enrich BPs and should be used in combination with other extraction and clean-up techniques to increase the recovery rates of target analytes.

### 4.2. Sample Preparation Techniques for BPs in HM and IF

#### 4.2.1. Liquid–Liquid Extraction (LLE)

LLE is one of the oldest and most common sample pre-treatments for extracting contaminants from food matrices. It is a versatile technique for separating, concentrating, or purifying components in a liquid mixture. LLE has been the most used technique for BP extraction in IF and HM, reported in 35% of the studies.

**Figure 9:**
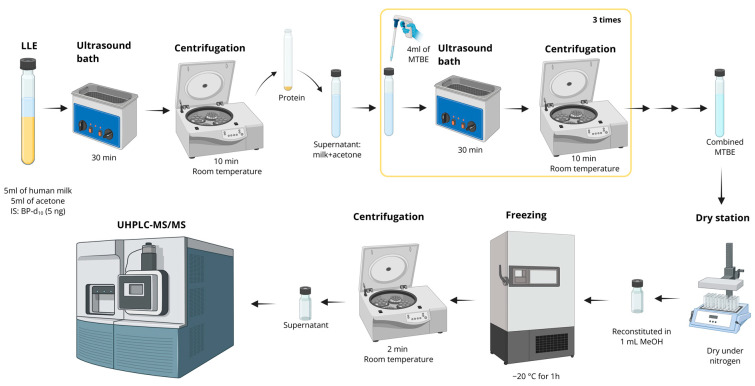
Liquid–liquid extraction using acetone for protein precipitation and methyl tert-butyl ether (MTBE) for photoiniators extraction, followed by LC-MS/MS detection. Protocol based on the method proposed by Liu & Mabury [13]. Created in BioRender. Oliveira, W. (2026) https://BioRender.com/fupqfl1.

The technique involves a separation process in which one or more substances are transferred from one liquid phase to another. The transference occurs due to differences in solubility between two immiscible phases, typically an aqueous phase and an organic phase. At equilibrium, the compounds partition preferentially into the phase with higher solubility, resulting in higher concentrations in that phase [75]. This method is also known as solvent extraction or partitioning and can be carried out in a single way or multiple steps (sequential), ensuring better extraction of the analyte. The disadvantage of LLE is the use of large volumes of solvents, which generate substantial amounts of chemical residues [76]. Figure 9 shows the flow of the LLE method by Liu & Mabury [13].

Acetonitrile, hexane, and dichloromethane, either individually or in combination with other solvents, have been the most commonly used solvents for the extraction of BPs [13,14,64]. Acetonitrile and acetone have also been used for both LLE and protein precipitation [13,63]. Furthermore, many researchers have used LEE in combination with other sample preparation techniques, such as SPE, SBSE, and dSPE [14,15,46,48,59,60,61]. Tran et al. [64] analyzed non-target compounds in breast milk samples from three donors in San Diego, USA. The extraction and cleaning method used were based on the combination of solvents (hexane/dichloromethane), silica, and SPE.

#### 4.2.2. Solid-Phase Extraction (SPE)

**Figure 10:**
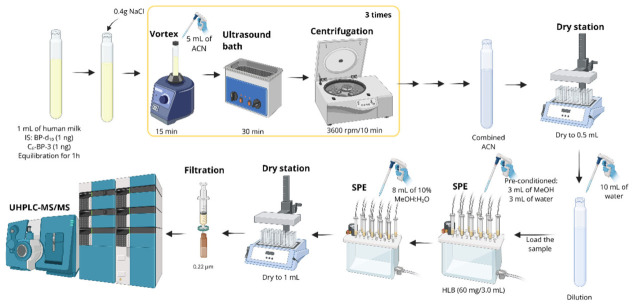
Schematic solid-phase extraction protocol using an HLB cartridge for the quantification of UV filters in human milk. Created in BioRender. Oliveira, W. (2026) https://BioRender.com/fupqfl1.

SPE has been used to complement or replace LLE and was reported in 35% of studies. The main objectives of SPE are to concentrate trace analytes, simplify the sample matrix by cleanup, and facilitate medium exchange by transferring analytes from the original sample matrix to a different solvent. The extraction consists of passing a sample stream of gas, fluid, or solvent through the SPE cartridge containing a suitable solid phase. The solid phase retains the analytes, which are later recovered by elution or thermal desorption [77]. Figure 10 shows an SPE protocol based on the method proposed by Sun et al. [15]. SPE can also be used to retain interferents that were not removed during previous sample-preparation steps.

The technique excels compared to LLE due to its shorter sample processing time and reduced solvent use. However, the most common problems with SPE have been the lack of reproducibility of some solid phases, as well as contamination from manufacturing materials used in cartridge packaging [77]. Moreover, traditional SPE (cartridges or pre-columns) requires multiple manual intervention steps. This drawback has been addressed through automation of sample preparation, such as on-line SPE, which improves the reproducibility of results [49,59].

On the other hand, considering the complexity of HM and IF, a major advantage of using SPE to analyze BPs in these matrices is the wide variety of available stationary phases, such as C18, C8, SAX, florisil, silica, among others, which facilitate effective extraction and matrix clean-up [49,51].

Among the stationary phases reported, octadecyl (C18) and hydrophilic-lipophilic balanced-HLB (divinylbenzene and vinylpyrrolidone-based) phases were the most used, followed by silica and primary-secondary amine (PSA) sorbents [68]. C18 is widely applied for retaining non-polar analytes, whereas HLB phases enable the extraction of a broad range of compounds, including the acidic, neutral, and basic species. Both phases, along with silica, have been extensively employed for the extraction of BPs and other target analytes [15,49,59,60,64,65]. In contrast, PSA have been mainly utilized for matrix clean-up, particularly for lipid removal. This clean-up step improves method sensitivity and reduces maintenance costs associated with analytical instrumentation.

#### 4.2.3. Dispersive Solid-Phase Extraction (dSPE)

In dispersive solid-phase extraction, the sorbent is directly dispersed into the sample solution, enabling rapid and efficient contact between the analytes or interferents and the sorbent material. This approach accelerates mass transference, reducing overall sample preparation time [78]. After adsorption, analytes retained on the sorbent can be recovered by solvent elution, thermal desorption, or directly quantified using an appropriate technique. For clean-up purposes, interfering compounds bound to the sorbent can be removed by centrifugation, leaving the purified sample matrix for subsequent analysis.

Anastassiades et al. [79] were the first to report the use of dispersed sorbents aimed at enhancing method selectivity. In this approach, unconditioned sorbent was added to the organic extract (acetonitrile) to remove sugars, pigments, fatty acids, and other interferents. The acronym QuEChERS was introduced to describe the method’s main features: quick, easy, cheap, effective, rugged, and safe. In many cases, the method is not applied to pre-concentrate target compounds. Regarding sample preparation time, the method has been shown in the literature to be slightly longer than LLE and SPE [80].

An advantage of dSPE is its ability to combine sorbents of different compositions and selectivities, thereby enhancing the coverage of target analytes recovered [81]. Furthermore, the technique is easy to perform, does not require specialized equipment, uses little solvent, and was reported in 20% of the studies.

Galindo et al. [48] evaluated BPs in IF using C18, PSA, and graphitized carbon black (GCB) in the clean-up step. Although GCB effectively removed matrix interferences, it also strongly retained planar compounds such as BP-12, thereby significantly reducing recovery rates. Following optimization, PSA was selected as the sole sorbent for clean-up. Similarly, Rodríguez-Gómez et al. [50] and Vela-Soria et al. [62] employed a mixture of PSA, C18, and MgSO_4_ for the clean-up of HM extracts in the evaluation of UV filters. This approach provided satisfactory performance for the monitoring of common UV filters in HM.

Other sorbents, such as zirconia-based materials (Z-Sep), have been reported to efficiently remove lipids and improve the recoveries of BPs and other contaminants, yielding better results than PSA and C18 [47].

#### 4.2.4. Low Temperature Partition Extraction (LTP)

Low-temperature partitioning extraction has emerged as an alternative and versatile methodology for the isolation of organic contaminants from complex matrices [82]. This technique has evolved over the years and is currently applied alongside other techniques, such as LLE and dSPE [83,84]. The LTP procedure consists of adding a small volume of a miscible organic solvent to an aqueous sample, followed by freezing the mixture for at least 3 h [82]. During this process, the aqueous phase solidifies, while the organic solvent containing the target analytes remains liquid. The freezing temperature applied is determined by the freezing point of the selected solvent.

The method allows an efficient cleanup of extracts containing significant amounts of lipids and proteins [85]. Acetonitrile has been widely used for LTP at −20 °C. However, other solvents and temperatures can be used if freezing is performed above the extracting solvent’s melting point.

The major limitation of this technique is the extended freezing time required, which can last up to 12 h. Two papers reported the use of LTP for BP extraction, as shown in Table 3 [48,64].

**Figure 11:**
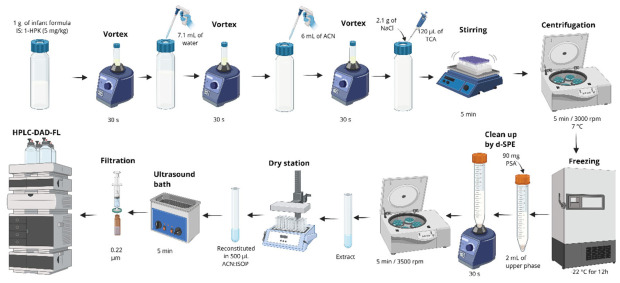
Sample preparation based on dispersive solid-phase extraction (dSPE) combined with low-temperature partition (LTP) for the extraction of benzophenones in infant formula, followed by HPLC-DAD-FL quantification. Created in BioRender. Oliveira, W. (2026) https://BioRender.com/fupqfl1.

The selection of the extraction solvent is a critical parameter in optimizing the LTPE method. Galindo, Oliveira and Godoy [48] evaluated acetonitrile, hexane, and dichloromethane for an LTP procedure. The authors reported that highly hydrophobic BPs exhibited greater losses during freezing, particularly when hexane or dichloromethane was used. This effect was attributed to the association of BPs with lipids and other apolar co-extractives from the IF, which segregated from the organic phase, resulting in lower recoveries. Beyond lipid removal, LTP also mitigated interferences arising from other compounds, such as starches and emulsifiers, added to infant formula or to formulations intended for infants with dysphagia. This approach enhanced method selectivity and demonstrated strong potential as a monitoring tool, given its simplicity, low cost, reduced waste generation, and overall analytical robustness. The protocol proposed by the authors is shown in Figure 11.

#### 4.2.5. Dispersive Liquid–Liquid Microextraction (DLLME)

Dispersive liquid–liquid microextraction was first introduced by Rezaee and colleagues for the determination of polycyclic aromatic hydrocarbons in water [86]. The technique has gained wide application due to its simplicity, low cost, high enrichment factor, and minimal consumption of organic solvents [87].

**Figure 12:**
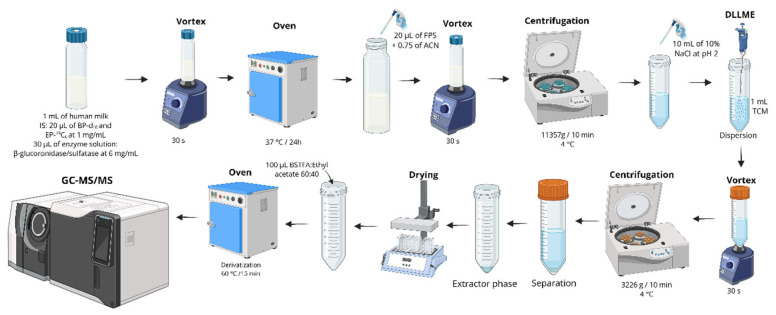
Schematic overview of the analytical workflow for the determination of bisphenols, parabens, and benzophenones in breast milk using fat/protein precipitation, followed by DLLME using trichloromethane (TCM), and subsequent derivatization prior to GC–MS/MS quantification. Created in BioRender. Oliveira, W. (2026) https://BioRender.com/fupqfl1.

DLLME is based on a ternary solvent system consisting of an extraction solvent, a disperser solvent miscible with both the extraction solvent and the aqueous phase, and the sample solution. To perform the extraction, a mixture of the extraction and disperser solvents is rapidly injected into the aqueous sample, producing a cloudy solution containing fine droplets of the extraction solvent. These droplets generate a large interfacial surface area, enhancing the partitioning of analytes into the extraction phase. Next, centrifugation is used to separate the system into two distinct phases, allowing straightforward collection of the enriched analytes. The DLLME protocol proposed by Iribarne-Durán et al. was shown in Figure 12 [66].

The main advantage of DLLME lies in the instantaneous dispersion of the extraction solvent, which maximizes surface area and accelerates analyte transfer. However, when low-density solvents are used, additional steps, such as freezing or the addition of surfactants, may be required to achieve complete phase separation, increasing analysis time. Moreover, incomplete separation between some dispersers and extraction solvents might limit the method’s efficiency, representing a drawback of DLLME [88,89].

Despite the high enrichment factors achievable with DLLME, which enable the detection of compounds at trace levels, only 20% of applications have employed this technique for the analysis of benzophenones in HM [51,66,67,69]. Three of them were applied after the enzymatic treatment of HM samples. In these cases, UV filters and endocrine-disrupting compounds, including BPs, were extracted using trichloromethane as the extractor and acetone as the disperser solvent.

#### 4.2.6. Stir Bar Sorptive Extraction (SBSE)

Introduced by Baltussen and co-workers, SBSE is a solventless sample preparation technique used for aqueous samples [90]. The technique is based on sorptive extraction, in which analytes are extracted using a magnetic stir bar coated with an adsorbent polymer, usually polydimethylsiloxane (PDMS) or PDMS/ethylene glycol. After extraction, the compounds can be eluted with a solvent or desorbed in a thermal desorption unit (TDU).

Currently, only two phases are commercially available, which limits the application of SBSE to the extraction of polar compounds. On the other hand, SBSE employs a relatively large volume of adsorbent, resulting in higher recoveries. Furthermore, the technique is fully automated and enables simultaneous extraction using multi-position stir plates, allowing high throughput [91].

The main factor influencing recovery in SBSE is the ratio of the partition coefficient and the phase ratio of PDMS on the stir bar to the sample [92]. Additionally, for the extraction of nonpolar analytes, an organic modifier is often required to reduce the adsorption of analytes onto container walls.

Rodríguez-Gómez et al. [61] applied this technique to detect contaminants in HM. They evaluated several parameters affecting SBSE, including extraction time, sample volume, solvent type, stirring speed, desorption solvent, and desorption time. The method provided satisfactory results and was successfully applied to determine target compounds in HM samples from 10 randomly selected women.

### 4.3. Instrumentation

Gas chromatography (GC), high-performance liquid chromatography (HPLC), and ultra-high-performance liquid chromatography (UHPLC) have been the main instruments used to analyze BPs in HM and Ifs (Table 3). However, liquid chromatography excels because it does not require derivatization prior to analysis.

With respect to detection systems, conventional UV and diode array detectors (DAD) have often been used for analyzing BPs in IFs. Their widespread use is due to their operational simplicity, the wavelength selectivity of BP derivatives, and lower cost compared with mass spectrometers. For example, wavelengths of 290 nm have been applied to monitor BP-1, BP-3, BP-8, and BP-12, while 250 nm has been used for BP [48]. However, in both IFs and HM, DAD can provide lower specificity and sensitivity compared to mass spectrometry. Similarly, fluorescence detectors are rarely used because not all molecules exhibit fluorescence, which limits the technique’s applicability. In such cases, an additional derivatization step is required [93].

Mass spectrometry (MS) is the most used technique for analyzing BPs in HM and IFs. The principle of the technique involves the conversion of the analytes into ions in the gas phase, which are measured through the mass/charge (*m*/*z*) ratio using mass analyzers that may operate with or without a chromatographic system [94]. The most common ionization sources are ESI+ for LC-MS and EI for GC-MS (Table 3). Triple quadrupole (QqQ), single quadrupole (Q), and time-of-flight (ToF) analyzers are the most frequently reported detectors in the literature for BP detection.

High-resolution mass spectrometry (HRMS) significantly enhances analytical performance by providing high mass accuracy and resolution. These features enable full-scan data acquisition and the simultaneous detection of a wide range of compounds, including those not initially targeted. Moreover, advanced mass analyzers, such as time-of-flight (qTOF) and Orbitrap, enable detailed characterization of both precursor ions and their fragments, facilitating the structural elucidation of unknown compounds. An additional advantage of HRMS is its application to suspect- and non-target-screening strategies, which support a more comprehensive characterization of sample chemical profiles. Consequently, HRMS represents a powerful complementary approach to targeted analytical methods, expanding detection capabilities and proving particularly valuable for the investigation of emerging contaminants, such as benzophenones and their derivatives [65,95,96]. However, its application remains constrained by challenges related to sample preparation and matrix complexity, as highlighted in the literature.

The main advantage of MS over classical chromatographic detectors (UV-Vis, DAD, fluorescence) is its high specificity. Monitoring is based on the *m*/*z* of each analyte, in combination with fragmentation patterns and chromatographic retention times. This allows accurate quantification even when complete chromatographic separation is not achieved [93].

## 5. Conclusions and Prospects for Analysis of Benzophenones

BP has gained attention in recent years due to its potential endocrine-disrupting effects, environmental persistence, and bioaccumulation. Infants can be exposed to BPs through various routes, including dermal absorption and ingestion via IF or HM. However, compared with other classes of chemical contaminants, the number of studies addressing the occurrence of BPs in HM and IF remains limited.

Bibliometric analysis has proven a valuable tool for assessing the quantitative growth and evolutionary trends of the literature on sample preparation techniques for BP quantification. Several studies have developed or adapted analytical methods to enable their determination in HM and IF.

Chromatographic methods coupled with mass spectrometry (GC-MS, GC-MS/MS, LC-MS, LC-MS/MS) remain the most frequently employed analytical techniques for BP quantification in these matrices. In contrast, high-resolution mass spectrometry (HRMS) improves analytical capabilities by providing high mass accuracy. These features enable full-scan data acquisition and the simultaneous detection of many compounds, including those not previously targeted. For this, both platforms, such as quadrupole time-of-flight (qTOF) and Orbitrap, allow the acquisition of detailed information on both precursor ions and their fragments at high resolution, contributing to the structural elucidation of unknown compounds. Another important advantage of HRMS is its application in suspect- and non-target-screening strategies, enabling comprehensive exploration of sample chemical profiles. Thus, HRMS stands out as a complementary approach to targeted techniques applied to biological samples and food for analyzing benzophenone and its derivatives, despite challenges related to sample preparation and the complexity of matrices, as discussed by the authors [65,95,96].

Nevertheless, there has been an increasing demand for greener and miniaturized sample preparation methods, driven by the need to reduce solvent consumption, simplify analytical workflows, and adhere to the principles of green chemistry. Over the past decade, Green Analytical Chemistry (GAC) principles have guided the development of sample preparation approaches for BPs analysis, with emphasis on minimizing the use of hazardous organic solvents while maintaining high analytical performance. In this context, techniques such as single-drop microextraction, nanomaterials, ionic liquids, and deep eutectic solvents (DES) have been successfully applied to environmental matrices [97,98,99,100]. These strategies can be applied individually or in combination, depending on the analytical objective. A significant challenge is selecting suitable green solvents and nanomaterials that match the physicochemical characteristics of HM and IF to optimize analyte recovery and sensitivity. Consequently, robust and fully validated green methodologies for BPs in baby food analysis remain scarce, as well as the application of HRMS. Nevertheless, this gap in the literature represents a valuable opportunity, as the continued integration of miniaturized extraction techniques with sustainable solvents and materials holds strong potential for the development of efficient, sensitive, and green analytical methods.

Given the potential risks BPs pose to human health and the environment, targeted studies are needed to refine analytical methods and thereby mitigate exposure in both adults and infants. Regulatory agencies play a crucial role in issuing guidelines and establishing limits to reduce BP contamination. It is important to note that breastfeeding should not be discouraged, even in the presence of BPs, as the benefits of breastfeeding outweigh the potential risks.

## Figures and Tables

**Table 1:** Benzophenone names, molecular structures, and chemical properties.

Name	Abbreviation	Formula	CAS	Chemical Structure	Molecular Weight (g/mol)	Log P ^a^	pKa ^a^
Benzophenone	BP	C_13_H_10_O	119-61-9	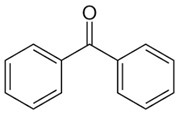	182.22	3.43	-
4-hydroxybenzophenone	4-OH-BP	C_13_H_10_O_2_	1137-42-4	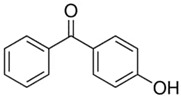	198.22	3.12	7.85
2,4-dihydroxybenzophenone	BP-1	C_13_H_10_O_3_	131-56-6	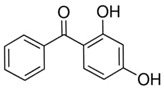	214.22	3.47	7.75
2,2′,4,4′-Tetrahydroxybenzophenone	BP-2	C_13_H_10_O_5_	131-55-5	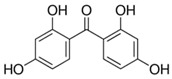	246.21	3.51	7.41
Oxybenzone	BP-3	C_14_H_12_O_3_	131-57-7	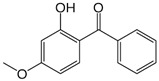	228.24	3.62	8.07
2,2′-Dihydroxy-4,4′-dimethoxybenzophenone	BP-6	C_15_H_14_O_5_	131-54-4	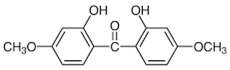	274.27	3.81	7.74
Dioxybenzone	BP-8	C_14_H_12_O_4_	131-53-3	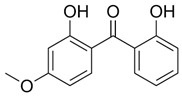	244.024	3.96	7.78
2-Hydroxy-4-(octyloxy)benzophenone	BP-12	C_21_H_26_O_3_	1843-05-6	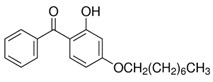	326.4	6.72	8.07
Methyl-2-(benzoyl)benzoate	MBB	C_15_H_12_O_3_	606-28-0	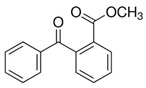	240.25	3.43	-
4-phenylbenzophenone	4-PBZ	C_19_H_14_O	2128-93-0	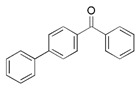	258.3	5.08	-
4-methylbenzophenone	4-MBP	C_14_H_12_O	134-84-9	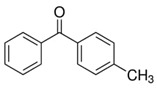	196.24	3.96	-
2,2-dimethoxy-2-phenylacetophenone	DMPA	C_16_H_16_O_3_	24650-42-8	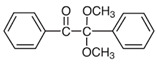	256.3	3.89	-
1-hydroxycyclohexyl phenyl ketone	HCPK	C_13_H_16_O_2_	947-19-3	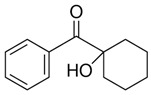	204.26	2.74	12.98

^a^: ChemAxon [6].

**Table 2:** Composition of mature human milk and infant formulas based on cow’s milk and soy.

Composition	IF—Cow’s Milk	g/100 kcal		IF- Soy	g/100 kcal		Human Milk	%
		Min	Max		Min	Max		
Protein	Casein and whey protein	1.8	3.0	Isolated protein of soy	2.25	3.0	Casein, α-lactalbumin, lactoferrin, secretory IgA immunoglobulin, lysozyme, and serum albumin	0.8 to 0.9
Lipids	Vegetable fat, dairy fat, fish oil	4.4	5.0	Vegetable fat	4.4	5.0	Palmitic and oleic acids, omega-3, and omega-6	3.0 to 5.0
Carbohydrates	Lactose, Maltose, Sucrose, glucose, Maltodextrin, glucose syrup, starches	9.0	14.0	Lactose, Maltose, Sucrose, glucose, Maltodextrin, glucose syrup, starches	9.0	14.0	Lactose and 30 or more oligosaccharides	6.9 to 7.2
Vitamins	A, D, E, K, C, B complex, etc.			A, D, E, K, C, B complex, etc.			A, B1, B2, B6, B12, D, K *	0.2
Minerals	Fe, Ca, Na, Mg, P, K, Se, Zn, etc.			Fe, Ca, Na, Mg, P, K, Se, Zn, etc.			Na, K, Ca, Mg, P, Cl	

* Vitamin K is found in negligible amounts in human milk.

**Table 4:** Leading scientific journals contributing to the analytical research of benzophenones in human milk and infant formula, ranked by total publication volume.

Journal	Number of Publications	Impact Factor *	Publisher	Zone
Journal of Chromatography A	4	4.0	Elsevier	1
Talanta	3	6.1	Elsevier	1
Environmental Science & Technology Letters	2	8.8	Amer Chemical Soc	2
Science of the Total Environment	2	8.0	Elsevier	2
Analytica Chimica Acta	1	6.0	Elsevier	2
Bioanalysis	1	1.8	Taylor & Francis Ltd.	2
Chemosphere	1	8.1	Pergamon-Elsevier Science Ltd.	2
Environmental Research	1	7.7	Academic Press Inc Elsevier Science	3
Environmental Science & Technology	1	11.3	Amer Chemical Soc	3
Journal of Dairy Science	1	4.4	Elsevier Science Inc	3
Reproductive Toxicology	1	2.8	Pergamon-Elsevier Science Ltd.	3
Separations	1	2.7	MDPI	3

* The Journal Impact Factor (JIF) was consulted in the Journal Citation Reports dataset from the Web of Science.

**Table 5:** Most globally cited documents on benzophenone analysis in infant matrices.

Author	Year	Title	Total Citations	Journal
Ye et al. [60]	2008	Automated on-line column-switching HPLC-MS/MS method with peak focusing for measuring parabens, triclosan, and other environmental phenols in human milk	219	Analytica Chimica Acta
Ye et al. [59]	2006	Measuring environmental phenols and chlorinated organic chemicals in breast milk using automated on-line column-switching–high performance liquid chromatography–isotope dilution tandem mass spectrometry	192	Journal of Chromatography B
Hines et al. [49]	2015	Concentrations of environmental phenols and parabens in milk, urine and serum of lactating North Carolina women	147	Reproductive Toxicology
Baduel et al. [47]	2015	Development of sample extraction and clean-up strategies for target and non-target analysis of environmental contaminants in biological matrices	122	Journal of Chromatography A
Molins-Delgado et al. [63]	2018	Determination of UV filters in human breast milk using turbulent flow chromatography and babies’ daily intake estimation	85	Environmental Research
Rodríguez-Gómez, Zafra-Gómez et al. [61]	2014	Gas chromatography and ultra high performance liquid chromatography tandem mass spectrometry methods for the determination of selected endocrine disrupting chemicals in human breast milk after stir-bar sorptive extraction	68	Journal of Chromatography A
Rodríguez-Gómez, Jiménez-Díaz et al. [14]	2014	A multiresidue method for the determination of selected endocrine disrupting chemicals in human breast milk based on a simple extraction procedure	55	Talanta
Rodríguez-Gómez et al. [50]	2015	Determination of benzophenone-UV filters in human milk samples using ultrasound-assisted extraction and clean-up with dispersive sorbents followed by UHPLC–MS/MS analysis	54	Talanta
Sanches-Silva et al. [46]	2008	Development of an analytical method for the determination of photoinitiators used for food packaging materials with potential to migrate into milk	42	Journal of Dairy Science
Iribarne-Durán et al. [66]	2022	Biomonitoring bisphenols, parabens, and benzophenones in breast milk from a human milk bank in Southern Spain	34	Science of The Total Environment

## Data Availability

The original contributions presented in this study are included in the Review/supplementary material. Further inquiries can be directed to the corresponding authors.

## Supplementary Materials

The following supporting information can be downloaded at: https://www.mdpi.com/article/10.3390/foods15101693/s1.
